# DEPTOR is an in vivo tumor suppressor that inhibits prostate tumorigenesis via the inactivation of mTORC1/2 signals

**DOI:** 10.1038/s41388-019-1085-y

**Published:** 2019-11-04

**Authors:** Xiaoyu Chen, Xiufang Xiong, Danrui Cui, Fei Yang, Dongping Wei, Haomin Li, Jianfeng Shu, Yanli Bi, Xiaoqing Dai, Longyuan Gong, Yi Sun, Yongchao Zhao

**Affiliations:** 1 Key Laboratory of Combined Multi-Organ Transplantation, Ministry of Public Health, First Affiliated Hospital, Zhejiang University School of Medicine, Hangzhou, China; 2 Institute of Translational Medicine, Zhejiang University School of Medicine, Hangzhou, China; 3 Cancer Institute of the Second Affiliated Hospital, Zhejiang University School of Medicine, Hangzhou, China; 4 Department of Oncology, Nanjing First Hospital, Nanjing Medical University, Nanjing, China; 5 Children’s Hospital, Zhejiang University School of Medicine, Hangzhou, Zhejiang China; 6 Department of Radiation Oncology, University of Michigan, Ann Arbor, MI USA

**Keywords:** Prostate cancer, TOR signalling, Cell migration

## Abstract

The DEPTOR-mTORC1/2 axis has been shown to play an important, but a context dependent role in the regulation of proliferation and the survival of various cancer cells in cell culture settings. The in vivo role of DEPTOR in tumorigenesis remains elusive. Here we showed that the levels of both DEPTOR protein and mRNA were substantially decreased in human prostate cancer tissues, which positively correlated with disease progression. DEPTOR depletion accelerated proliferation and survival, migration, and invasion in human prostate cancer cells. Mechanistically, DEPTOR depletion not only activated both mTORC1 and mTORC2 signals to promote cell proliferation and survival, but also induced an AKT-dependent epithelial–mesenchymal transition (EMT) and β-catenin nuclear translocation to promote cell migration and invasion. Abrogation of mTOR or AKT activation rescued the biological consequences of DEPTOR depletion. Importantly, in a *Deptor*-KO mouse model, *Deptor* knockout accelerated prostate tumorigenesis triggered by *Pten* loss via the activation of mTOR signaling. Collectively, our study demonstrates that DEPTOR is a tumor suppressor in the prostate, and its depletion promotes tumorigenesis via the activation of mTORC1 and mTORC2 signals. Thus, DEPTOR reactivation via a variety of means would have therapeutic potential for the treatment of prostate cancer.

## Introduction

Prostate cancer is one of the most common cancers, and the second leading cause of death among male cancer patients [1]. The progression of prostate cancer includes intraepithelial neoplasia, adenocarcinoma in situ, invasive carcinoma, and metastasis [2]. Radiotherapy and hormone therapy are commonly served as primary treatments for patients with prostate cancer. However, a considerable proportion of patients respond poorly to both therapies and then progress to metastatic disease [3, 4]. Therefore, more novel and effective targeted therapies are greatly in high demand. Due to the diverse genomic aberrations in prostate cancer, multiple targeted therapies are being investigated for advanced prostate cancer, such as androgen receptor antagonists [5], AKT inhibitors [6], and PARP inhibitors [7]. Thus, a better understanding of the mechanisms underlying the genesis and progression of prostate cancer would facilitate the discovery of novel targeted therapies that are effective for a significant number of patients.

Genetic alterations in the phosphoinositide 3-kinase (PI3K)/AKT pathway frequently occur in prostate cancer, which mediates tumor maintenance and progression [8]. Previous studies have demonstrated that the hyperactivation of the PI3K/AKT pathway appeared in almost all advanced prostate cancers, mainly occurring through the loss of PTEN [8–11], which results in resistance to hormone therapy and poor prognosis [12]. Therefore, developing novel targets against the PI3K/AKT pathway has great application potential for PTEN-deficient prostate cancer.

Mammalian target of rapamycin (mTOR) is an evolutionarily conserved serine/threonine protein kinase that belongs to the PI3K/AKT/mTOR pathway. It plays crucial roles in integrating both intracellular and extracellular signals, thus regulating cell growth, proliferation, survival, autophagy, and metabolism [13–15]. It is well known that mTOR forms two complexes that are distinctive in structure and function, namely, mTORC1 and mTORC2 [13–15]. Upon stimulation with growth factors, nutrients, and other stresses, mTORC1 functions as the central regulator of the signaling hub and mainly promotes cell growth and protein translation by phosphorylating S6K1 and 4E-BP1 [16]. Unlike mTORC1, mTORC2 regulates metabolism, survival, cytoskeletal organization, and cell mobility, mainly through activating AKT by phosphorylation at serine 473 [13–15]. Loss of either mTOR or AKT significantly suppresses prostate tumorigenesis in a PTEN-deficient model, suggesting that mTOR pathway is an attractive target for cancer treatment [17, 18]. Indeed, mTORC1 inhibitors, rapamycin and its analogs, such as everolimus, have been approved as anticancer agents [19, 20].

DEPTOR, a common component found in both the mTORC1 and mTORC2 complexes, directly binds to mTOR to block the activities of both complexes [21]. As a naturally occurring inhibitor of both complexes, DEPTOR functions as a putative tumor suppressor by suppressing protein synthesis, cell growth, proliferation, and survival. Paradoxically, DEPTOR acts as an oncoprotein under certain circumstances [16, 21, 22]. DEPTOR overexpression in multiple myeloma [21], as well as in hepatocellular carcinoma and thyroid carcinoma, is correlated with poor prognosis [23, 24]. Therefore, defining the role of DEPTOR in specific cancer types, and whether it is acting as a tumor suppressor or an oncoprotein, would help to uncover the mechanism underlying tumorigenesis and tumor progression; thus, it would aid in the design of personalized treatments for cancer patients.

In this study, we report that DEPTOR expression was significantly decreased in human prostate cancer tissues at both the protein and mRNA levels, and positively correlated with prostate cancer progression. DEPTOR depletion promoted proliferation, survival, cell migration, and invasion of prostate cancer cells, as a result of mTORC1 and mTORC2 activation. More importantly, the suppressive role of DEPTOR in prostate tumorigenesis was further confirmed using a *Deptor-*knockout (KO) mouse model in combination with a heterozygous *Pten* deletion. Thus, DEPTOR inhibits prostate tumorigenesis by inactivating mTOR signals, suggesting the potential use of mTOR inhibitors to treat prostate cancer with low DEPTOR expression.

## Results

### DEPTOR expression is decreased in human prostate cancer tissues

**Fig. 1:**
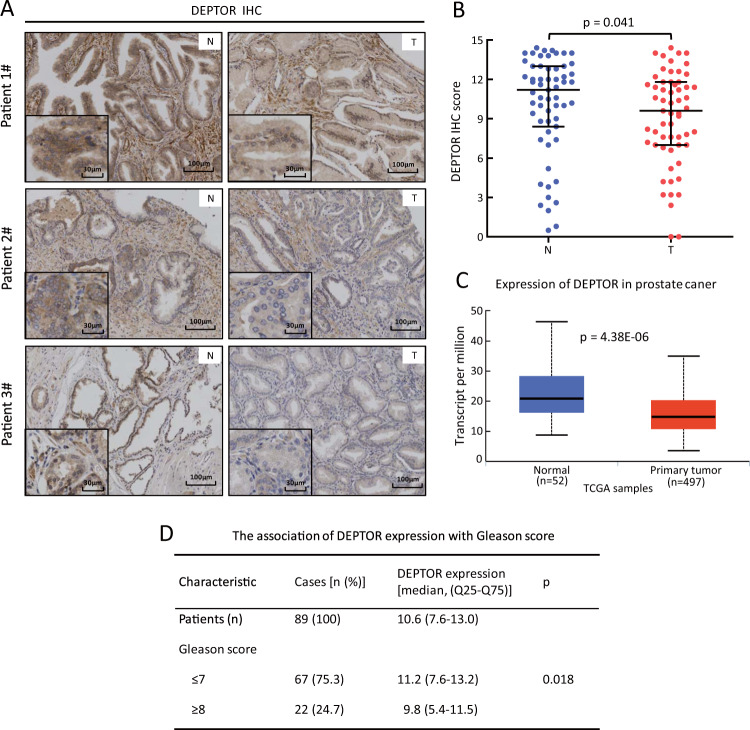
DEPTOR expression is decreased in prostate tumor tissues compared with their corresponding tumor-adjacent normal tissues. **a**, **b** The expression of DEPTOR protein is significantly reduced in human prostate cancer tissues. Prostate tissue microarrays containing 59 tumor tissues (T) and their corresponding tumor-adjacent normal tissues (N) were stained for DEPTOR expression. Representative images of DEPTOR staining are shown (**a**). Scale bars: 100 μm. Each IHC picture is shown in better resolution at the lower left corner. Scale bars: 30 μm. DEPTOR expression was evaluated using the IRS system according to the staining intensity and the percentage of positive cells (**b**). The Wilcoxon rank sum test was utilized to assess the DEPTOR expression from 59 pairs of prostate tissue samples. *p* = 0.041. **c** DEPTOR transcript is significantly reduced in primary prostate tumors compared with normal tissues from TCGA database. *p* < 0.001. **d** The association of DEPTOR expression in prostate tumor tissues from 89 patients with Gleason score

As a natural inhibitor of mTORC1 and mTORC2, DEPTOR is generally considered as a tumor suppressor, which promotes protein synthesis, cell growth and survival. However, DEPTOR could act as an oncogene under certain circumstances due to its relief of feedback inhibition to PI3K [16, 21, 22]. To explore the specific role of DEPTOR in prostate tumorigenesis, we examined potential alterations of DEPTOR expression in prostate tumor tissues compared with adjacent normal tissues. We first examined the specificity of DEPTOR antibody in immunohistochemistry (IHC) assay, using a blocking peptide (Supplementary Fig. 1A). Our result clearly showed that the blocking peptide significantly reduced the DEPTOR staining in human prostate cancer tissues (Supplementary Fig. 1A). The antibody specificity was further confirmed by complete lack of DEPTOR staining in prostate cancer cells transfected with sgDEPTOR (Supplementary Fig. 1B), and in vivo xenografts, derived from cells with shDEPTOR transfection (Supplementary Fig. 1C). Thus, this DEPTOR antibody has good specificity and suitable for IHC staining of human cell and tissue samples. Next, we performed immunostaining with this antibody on prostate tissue microarrays, which consist of 59 pairs of tumor and tumor-adjacent normal tissues. Based on the staining intensity and the percentage of cells with positive staining, tissues were classified into four groups (0, negative; 1, weak; 2, moderate; and 3, strong staining) for the intensity score and five groups (0, 0%; 1, ≤10%; 2, 11–50%; 3, 51–80% and 4, ≥81%) for the percentage score. The IHC score for DEPTOR was calculated by multiplying the percentage score by the intensity score. In 40 out of all 59 cases, DEPTOR expression was lower in tumor tissues (40/59, 67%) than in corresponding tumor-adjacent normal tissues (Fig. 1a). Statistical analysis by Wilcoxon rank sum test between the IHC scores of prostate tumor tissues and corresponding tumor-adjacent normal tissues showed that DEPTOR expression was significantly decreased in prostate cancer (*p* = 0.041) (Fig. 1b). Consistently, the statistical analysis of TPM (transcripts per million) data obtained from TCGA database [25], consisting of 497 prostate tumor tissues and 52 normal tissues, showed that DEPTOR expression at the mRNA level was also significantly decreased in prostate cancer tissues, compared with that of normal tissue (*p* < 0.001) (Fig. 1c). Moreover, lower DEPTOR levels were shown in tumor tissues with high Gleason score (*n* = 22, Gleason score ≥ 8) from 89 prostate cancer patients, whereas higher DEPTOR levels were shown in the tissues with low Gleason score (*n* = 67, Gleason score ≤ 7) (*p* = 0.018) (Fig. 1d), suggesting that the decrease of DEPTOR expression is associated with prostate cancer progression. Taken together, DEPTOR reduction in prostate cancer tissues and its correlation with disease progression suggest that reduced DEPTOR expression could contribute to the development of human prostate cancer.

### DEPTOR depletion promotes cell proliferation and survival, and activates mTORC1/2 signals in prostate cancer cells

**Fig. 2:**
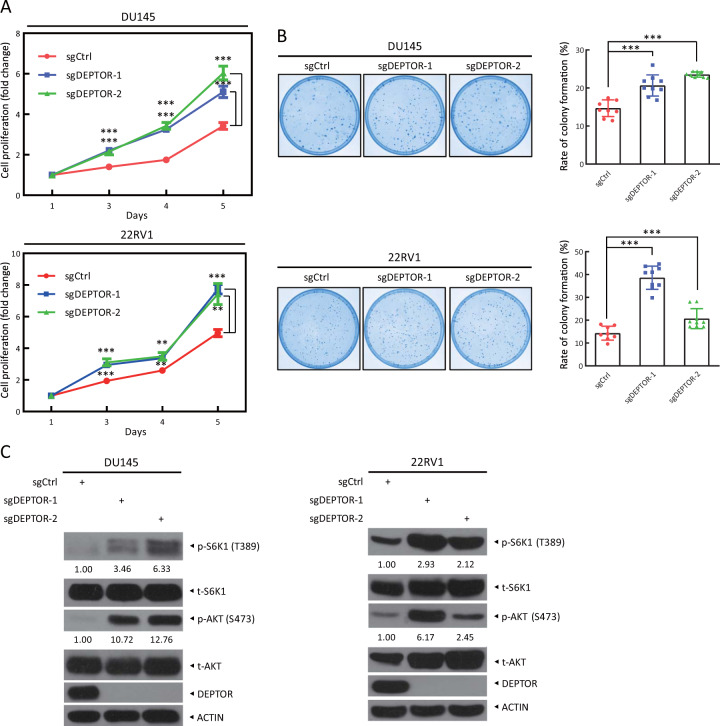
DEPTOR knockout promotes cell proliferation and clonogenic survival and the activation of S6K1 and AKT in prostate cancer cells. **a** DEPTOR knockout promotes the proliferation of prostate cancer cells. DEPTOR-knockout DU145 and 22RV1 cells were generated via CRISPR/Cas9 technology. Cells were seeded in 96-well plates in triplicate and then subjected to an ATPlite-based cell proliferation assay. Cell proliferation is expressed as the fold change compared with that at day 1. The mean ± SEM are shown from three independent experiments, *n* = 3; **p < 0.01; ***p < 0.001. **b** DEPTOR knockout promotes clonogenic survival of prostate cancer cells. Cells were seeded in 60-mm dishes at 500 cells per dish in triplicate and were incubated for 7–14 days, followed by staining (left) and colony counting (right). The mean ± SEM are shown from three independent experiments; *n* = 3; ***p < 0.001. **c** DEPTOR knockout causes increased phosphorylation of the mTORC1 and mTORC2 downstream effectors, S6K1 and AKT, respectively. Cells were harvested for western blotting using the indicated antibodies. The band density was quantified and expressed as the relative gray value (compared with the control), by arbitrarily setting the control value as 1

Given that DEPTOR expression is decreased in human prostate cancer tissues, we next determined the role of DEPTOR in the proliferation and survival of prostate cancer cells. First, we measured the levels of DEPTOR in multiple prostate cancer cells and found that DU145 and 22RV1 cells have high expression of DEPTOR and low phosphorylation of S6K1 and AKT (Supplementary Fig. 2). Interestingly, although 22RV1, Ala-41, DU145, and LNCap cell lines express comparable levels of DEPTOR, their levels of AKT and S6K1 phosphorylation are variable (Supplementary Fig. 2), likely due to different PTEN status in these cells (e.g., 22RV1 harboring *PTEN*^*+/+*^, DU145 harboring *PTEN*^*+/*^^−^, whereas LNCap harboring *PTEN*^*−/−*^) [26]. Thus, we next chose DU145 and 22RV1 cells to generate DEPTOR depleted cells via a CRISPR/Cas9-based approach and found that DEPTOR depletion significantly promoted cell proliferation in two individual colonies of sgDEPTOR cells in both prostate cancer cell lines, compared with that in sgCtrl cells (Fig. 2a). Likewise, the clonogenic formation assay indicated that DEPTOR depletion also significantly enhanced cell survival, as evidenced by higher rate of colony formation in sgDEPTOR cells (Fig. 2b). Moreover, increased phosphorylation of S6K1 and AKT, the downstream effectors of the mTORC1 and mTORC2 complexes [13, 16], respectively, was observed in sgDEPTOR cells (Fig. 2c). Further, DEPTOR knockdown by siRNA oligos targeting DEPTOR promoted cell proliferation and clonogenic survival in both DU145 and 22RV1 cells (Supplementary Fig. 3A, B). Compared with complete DEPTOR depletion on cell proliferation, the effect of silencing of DEPTOR by siRNA oligos decreased to a lesser extent (Supplementary Fig. 3A, B) and at later time points (Supplementary Fig. 3A), likely due to the incomplete depletion of the DEPTOR protein (Supplementary Fig. 3C). Consistently, the phosphorylation of S6K1 and AKT was also increased upon DEPTOR knockdown (Supplementary Fig. 3C). Taken together, our results suggest that DEPTOR depletion or knockdown promotes the proliferation and survival of prostate cancer cells, which is accompanied by the activation of mTORC1 and mTORC2 signals.

### Torin-1, a dual inhibitor of mTORC1 and mTORC2, rescues the proliferation-promoting effect of DEPTOR depletion

**Fig. 3:**
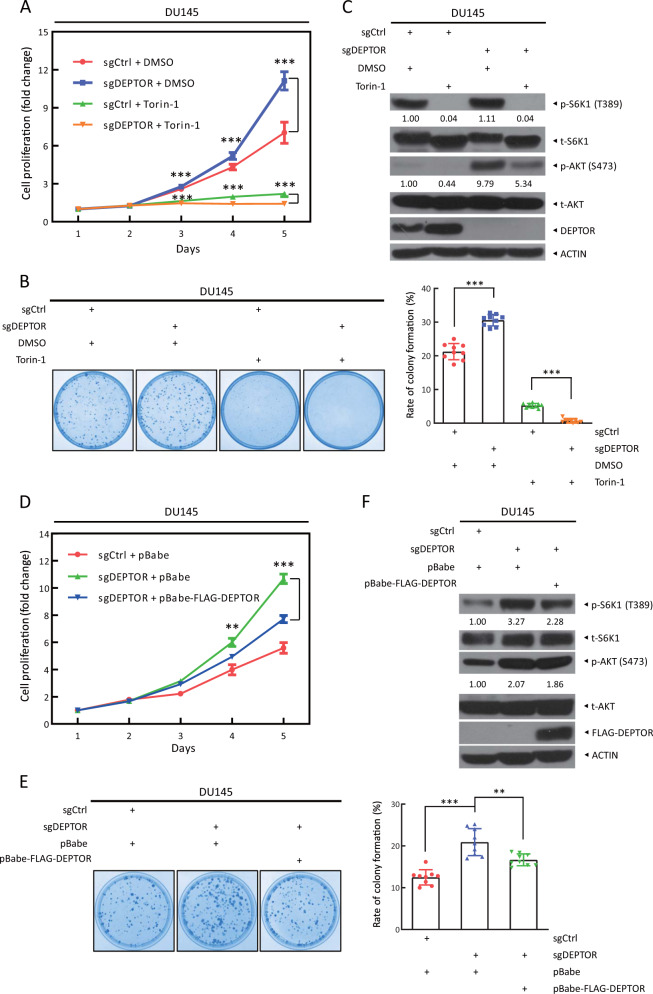
The cell proliferation and survival induced by DEPTOR knockout are abrogated by mTOR inhibitor Torin-1 and exogenous DEPTOR. **a** Torin-1 treatment abrogates the induction of cell proliferation by DEPTOR knockout. DU145 cells were seeded in 96-well plates in triplicate. Cells were treated with Torin-1 (100 nM) or were left untreated and were subjected to an ATPlite-based cell proliferation assay. Cell proliferation is expressed as the fold change compared with that at day 1. The mean ± SEM are shown from three independent experiments; *n* = 3; ***p < 0.001. **b** Torin-1 treatment abrogates the increased colony survival upon DEPTOR knockout. Cells were seeded in 60-mm dishes at 500 cells per dish and treated with DMSO or Torin-1 the following day. After 7–14 days, cell colonies were stained (left) and counted (right). The mean ± SEM are shown from three independent experiments; *n* = 3; ***p < 0.001. **c** mTOR activation upon DEPTOR knockout is blocked by Torin-1 treatment. Cells were treated with DMSO or Torin-1 for 12 h, followed by western blotting. **d** Exogenous DEPTOR expression abrogates the induction of cell proliferation by DEPTOR knockout. DU145 cells were infected with retrovirus expressing mock vector or DEPTOR, and then selected for stable expression with puromycin. Cells were seeded in 96-well plates in triplicate and then subjected to an ATPlite-based cell proliferation assay. Cell proliferation is expressed as the fold change compared with that at day 1. The mean ± SEM are shown from three independent experiments; *n* = 3; **p < 0.01; ***p < 0.001. **e** Exogenous DEPTOR expression abrogates the increase in colony survival upon DEPTOR knockout. Cells were seeded in 60-mm dishes at 500 cells per dish. After 7–14 days, cell colonies were stained (left) and counted (right). The mean ± SEM are shown from three independent experiments; *n* = 3; **p < 0.01; ***p < 0.001. **f** mTORC1 activation upon DEPTOR knockout is blocked by exogenous DEPTOR expression. Cells were harvested for western blotting using the indicated antibodies. The band density was quantified and expressed as the relative gray value (compared with the control), by arbitrarily setting the control value as 1

We next investigated whether the activation of mTORC1/2 signals plays a causal role in enhanced cell proliferation and survival by DEPTOR depletion. We treated DU145 cells with Torin-1, a synthetic small molecule inhibitor of mTORC1 and mTORC2 [27, 28]. We first determined that the lowest concentration of Torin-1 that significantly reduced the phosphorylation of S6K1 and AKT as readout for inhibition of mTORC1 and mTORC2, was 100 nM in DU145 cells (Supplementary Fig. 4). We then treated sgDEPTOR cells with Torin-1 and observed significantly decreased cell proliferation and clonogenic survival (Fig. 3a, b), indicating a rescue effect of Torin-1. Meanwhile, the immunoblotting result confirmed that Torin-1 indeed inactivated mTORC1 and mTORC2, as reflected by reduced phosphorylation of S6K1 and AKT (Fig. 3c).

Finally, we performed a rescue experiment to exclude possible off-target effects of sgDEPTOR due to CRISPR/Cas9 technology. We infected sgDEPTOR cells with retrovirus expressing DEPTOR and found that stably expressing exogenous DEPTOR significantly blocked the promotion of cell proliferation and survival by DEPTOR depletion (Fig. 3d, e). Meanwhile, ectopic DEPTOR expression also inactivated mTORC1, as reflected by reduced phosphorylation of S6K1 (Fig. 3f). However, it had no effect on the activation of mTORC2, as reflected by no change of AKT phosphorylation. Notably, Torin-1 treatment totally blocked S6K1 phosphorylation, but moderately reduced AKT phosphorylation (Fig. 3c), which is likely due to the relief of feedback inhibition to PI3K. Collectively, these results indicated that activation of mTORC1 and mTORC2 by DEPTOR depletion plays a causal role in the promotion of cell proliferation and survival.

### DEPTOR deletion enhances cell migration and invasion in prostate cancer cells

**Fig. 4:**
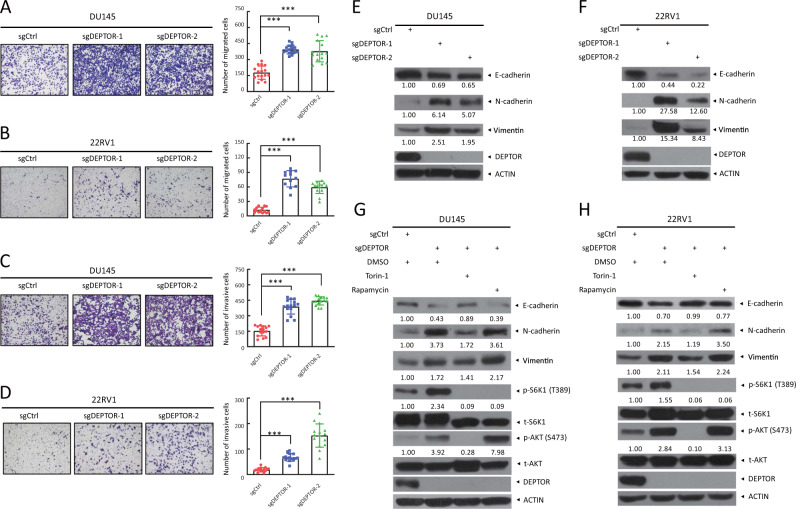
DEPTOR knockout enhances cell migration and invasion in prostate cancer cells, and Torin-1 abrogates the alterations of EMT markers upon DEPTOR depletion. **a, b** DEPTOR knockout promotes the migration of prostate cancer cells. DU145 (**a**) and 22RV1 cells (**b**) were transfected with the indicated sgRNA and then subjected to transwell migration assays. Representative images of migratory cells at 12–24 h are shown (left, **a** and **b**). The number of migratory cells was counted in five random fields per chamber insert (right, **a** and **b**). The mean ± SEM from three independent experiments are shown; *n* = 3; ***p < 0.001. **c**, **d** DEPTOR knockout promotes the invasion of prostate cancer cells. Cells were seeded into a 24-well plate chamber insert with Matrigel matrix and incubated for 12–24 h, followed by imaging (left). The invasive cells in five random fields per chamber insert were counted (right). The mean ± SEM from three independent experiments are shown; *n* = 3; ***p < 0.001. **e**, **f** DEPTOR knockout induces EMT in prostate cancer cells. DU145 (**e**) and 22RV1 (**f**) cells transfected with the indicated sgRNA were subjected to western blotting using the indicated antibodies. **g, h** Torin-1, but not rapamycin rescues the alterations of EMT markers upon DEPTOR depletion. DU145 (**g**) and 22RV1 (**h**) cells were treated with Torin-1 or rapamycin for 12 h and then subjected to western blotting using the indicated antibodies. The band density was quantified and expressed as the relative gray value (compared with the control), by arbitrarily setting the control value as 1

Previous studies have shown that DEPTOR levels are reduced in metastatic cancer cells (e.g., endometrial cancer tissues and breast cancer cells), and DEPTOR reduction or depletion promotes EMT (epithelial–mesenchymal transition), whereas its overexpression represses this process [22]. We next explored whether DEPTOR depletion had any effects on cell migration and invasion in this context, since aberrant activation of the PI3K/AKT pathway in cancers not only promotes cell growth and proliferation but also leads to tumor metastasis via promoting EMT during prostate cancer progression [29]. Indeed, DEPTOR depletion or knockdown (Supplementary Fig. 5A) promoted cell migration, as evidenced by the increase of migratory cells in the transwell migration assay (Fig. 4a, b, and Supplementary Fig. 5B), and a faster wound healing (Supplementary Fig. 5C, D). Consistently, cell invasion detected by the transwell invasion assay was also increased upon DEPTOR depletion (Fig. 4c, d, and Supplementary Fig. 5E).

To investigate the potential molecular mechanisms by which DEPTOR regulates cell migration and invasion, we measured potential alterations of EMT markers, including E-cadherin, a well-characterized epithelial marker, and N-cadherin and Vimentin, two well-known mesenchymal markers [30, 31]. Consistent with enhanced cell migration and invasion, decreased levels of E-cadherin and increased levels of N-cadherin and Vimentin were found upon DEPTOR depletion (Fig. 4e, f). Thus, cell migration and invasion stimulated by DEPTOR depletion are likely due to the enhanced EMT in prostate cancer cells. To determine whether the enhancement of EMT is caused by mTORC1 or mTORC2 activation upon DEPTOR depletion, we treated cells with Torin-1 (inhibiting both mTORC1 and mTORC2) or rapamycin (only inhibiting mTORC1) and tested the alterations of EMT markers. We found that the decreased E-cadherin and increased N-cadherin and Vimentin upon DEPTOR depletion were abrogated by Torin-1, but not by rapamycin (Fig. 4g, h). Collectively, these results strongly suggest that EMT is promoted by mTORC2 activation upon DEPTOR depletion.

### AKT inhibitor MK-2206 rescues migration-promoting effect of DEPTOR depletion

**Fig. 5:**
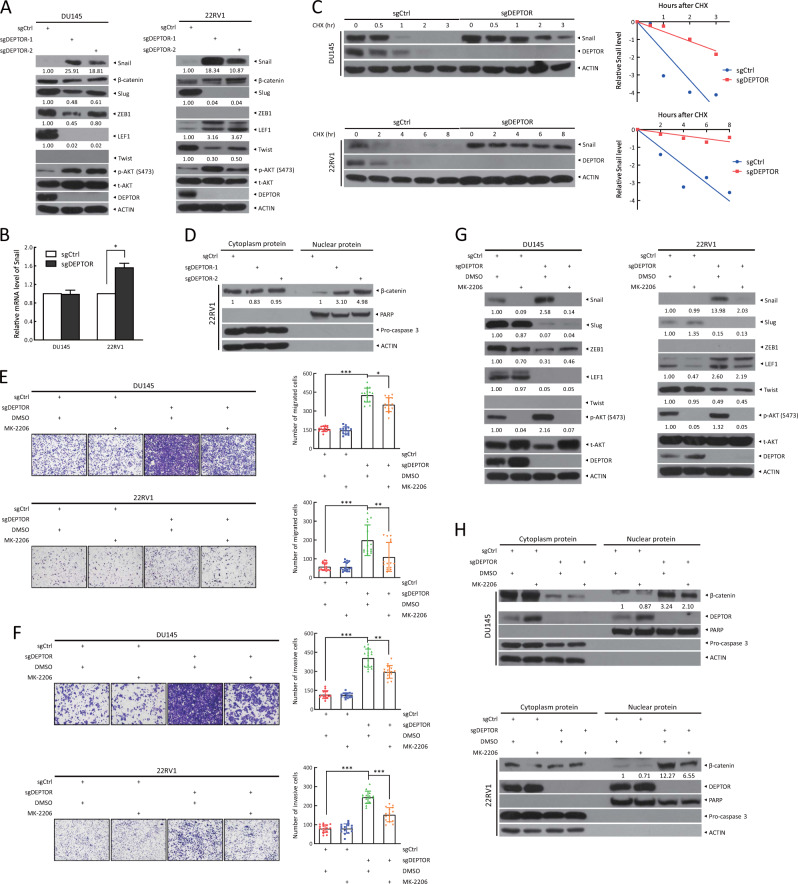
The induction of cell migration and invasion by DEPTOR depletion is abrogated by the AKT inhibitor MK-2206. **a** DEPTOR knockout causes the accumulation of Snail. Cells transfected with the indicated sgRNA were subjected to western blotting using the indicated antibodies. **b**, **c** DEPTOR knockout has minor effects on Snail mRNA levels, but extends Snail protein half-life. Cells were harvested for RT-qPCR analysis (**b**), or treated with CHX for various time periods, followed by western blotting (**c**). Densitometry quantification was performed with Image J, and the decay curves are shown (right, **c**). **d** DEPTOR knockout increases nuclear translocation of β-catenin. Cells transfected with the indicated sgRNA were subjected to nuclear fractionation, followed by western blotting using the indicated antibodies. PARP and Pro-caspase 3 served as markers of the nuclear and cytoplasmic fractions, respectively. **e**, **f** MK-2206 treatment abrogates the induction of cell migration and invasion by DEPTOR depletion. Cells were treated with MK-2206 or left untreated, and then subjected to transwell migration (**e**) and invasion (**f**) assays. Representative images of migratory and invasive cells are shown (left, **e** and **f**). The number of migratory and invasive cells was counted in five random fields per chamber insert (right, **e** and **f**). The mean ± SEM from three independent experiments are shown; *n* = 3; *p < 0.05, **p < 0.01; ***p < 0.001. **g** The induction of Snail upon DEPTOR knockout is reversed by MK-2206 treatment. Cells were treated with MK-2206 for 12 h or left untreated, followed by western blotting. **h** β-catenin nuclear translocation induced by DEPTOR knockout is partially inhibited by MK-2206 treatment. Cells were treated with MK-2206 or left untreated and then subjected to nuclear fractionation, followed by western blotting. The band density was quantified and expressed as the relative gray value (compared with the control), by arbitrarily setting the control value as 1

The expression of epithelial markers is negatively regulated by several transcriptional repressors, including the Snail family, ZEB family, LEF-1, and Twist [30, 31]. Previous studies showed that inhibition of AKT and mTORC2 decreased Snail expression to induce E-cadherin transcription, thus suppressing EMT [32, 33]. To investigate how DEPTOR affects E-cadherin expression, we detected the upstream transcriptional regulators of E-cadherin upon DEPTOR depletion. Compared with sgCtrl cells, Snail levels were significantly increased in DU145 and 22RV1 sgDEPTOR cells, accompanying with increased levels of phosphorylated AKT (Fig. 5a). However, upon DEPTOR depletion, the levels of Slug, ZEB1, and LEF-1 were significantly decreased and Twist was undetectable in DU145 cells; the levels of Slug and Twist were significantly decreased, LEF-1 levels were significantly increased, and ZEB1 was undetectable in 22RV1 cells. Taken together, these results suggest that the increase of Snail likely plays a major role in promoting EMT in prostate cancer cells upon DEPTOR depletion. Mechanistically, DEPTOR depletion had minor, if any, effects on the mRNA levels of Snail (Fig. 5b), but significantly extended the protein half-life of Snail (Fig. 5c), which may result from the activation of mTORC2, which can block GSK3-dependent Snail degradation [34, 35].

In addition, it has been previously shown that AKT could trigger nuclear translocation of β-catenin, a critical regulator of cancer cell invasion, to transactivate the expression of downstream target genes, thus promoting cell migration and invasion [30]. Indeed, we found that the levels of nuclear β-catenin were remarkably increased upon DEPTOR depletion (Fig. 5d). These data suggest that AKT activation upon DEPTOR depletion induces Snail expression on one hand, and triggers β-catenin nuclear translocation on the other hand.

We next used MK-2206, an allosteric inhibitor of AKT, to determine whether AKT activation indeed plays a causal role in enhanced migration and invasion by DEPTOR depletion. Indeed, MK-2206 partially abrogated the promoting effect of DEPTOR depletion in cell migration and invasion (Fig. 5e, f). Mechanistically, MK-2206 reversed Snail induction (Fig. 5g) and β-catenin nuclear translocation in both DU145 and 22RV1 cells (Fig. 5h). However, MK-2206 had minor, if any, effects on the levels of Slug, ZEB1, LEF-1, and Twist upon DEPTOR depletion, suggesting that alterations of these proteins upon DEPTOR depletion are independent on AKT activation (Fig. 5g). Taken together, these results demonstrated that AKT activation plays a causal role in migration-promoting effect of DEPTOR depletion by inducing Snail expression and β-catenin nuclear translocation.

### Deptor disruption induces prostate tumorigenesis via the activation of mTORC1 and mTORC2 signals in vivo

Having identified the reduction of DEPTOR expression in human prostate cancer tissues and its important role in the proliferation, survival, migration, and the invasion of prostate cancer cells in cell culture settings, we next determined whether DEPTOR depletion played a causal role in in vivo prostate tumorigenesis, triggered by heterozygous deletion of PTEN. To this end, we generated a *Deptor*-KO mouse model by Cre-driven deletion of exons 6 and 7 of the *Deptor* allele (Supplementary Fig. 6A), leading to removal of the PDZ domain responsible for the interaction with mTOR [21]. Thus, this *Deptor*-KO strain can be used as a physiological model to investigate mTOR-dependent functions of DEPTOR, although it remains to express an N-terminal portion of Deptor with 2 DEP domains with some possible, but unknown mTOR-independent functions. Mice with three *Deptor* genotypes (*Deptor*^*+/+*^, *Deptor*^*+/−*^, and *Deptor*^*−/−*^) were identified and confirmed by PCR genotyping (Supplementary Fig. 6B). Western blotting also confirmed a partial and complete elimination of Deptor protein in *Deptor*^*+/−*^ and *Deptor*^*−/−*^ mice, respectively (Supplementary Fig. 6C). The genotyping of 356 offspring at the age of 5 weeks or older from *Deptor*^*+/−*^ mice intercrossing revealed that the ratio of *Deptor*^*+/+*^:*Deptor*^*+/−*^:*Deptor*^*−/−*^ was ~1:2:1 (Supplementary Fig. 6D), suggesting that *Deptor* is dispensable to embryonic development. Meanwhile, the size (Supplementary Fig. 6E) and the body weight (Supplementary Fig. 6F) of the mice at the age of ~3 weeks among the three genotypes showed no significant difference, which is consistent with the findings using whole-body *Deptor*-KO mice with the deletion at the exon 2 [36]. These results indicate that *Deptor* disruption does not cause embryonic and postnatal death.

**Fig. 6:**
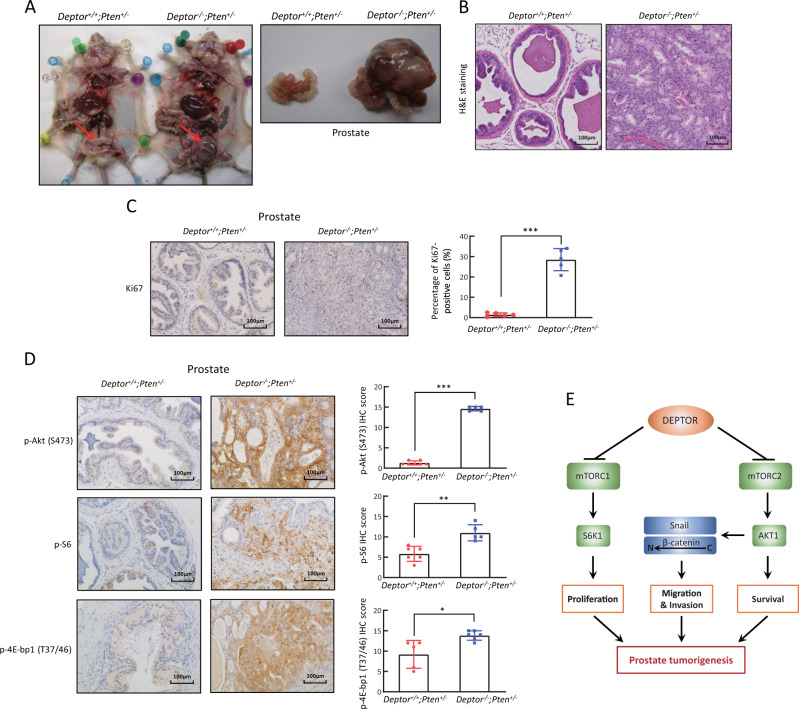
*Deptor* knockout induces prostate tumorigenesis triggered by *Pten* heterozygous loss via the activation of mTORC1 and mTORC2 signaling in mice. **a** *Deptor* knockout causes prostate tumorigenesis triggered by *Pten* heterozygous loss. Representative images of the mice and prostate glands from three pairs of *Deptor*^*+/+*^*;Pten*^*+/−*^ and *Deptor*^*−/−*^*;Pten*^*+/−*^ littermate mice. **b**, **c** Excessive proliferation of epithelial cells in prostate cancer tissues from *Deptor*^*−/−*^*;Pten*^*+/−*^ mice. The prostate tissues from *Deptor*^*+/+*^*;Pten*^*+/−*^ and *Deptor*^*−/−*^*;Pten*^*+/−*^ mice were sectioned and subjected to H&E staining (**b**) and Ki67 staining (**c**). **d** *Deptor* deletion activates mTORC1 and mTORC2 signaling during prostate tumorigenesis. The prostate tissues from *Deptor*^*+/+*^*;Pten*^*+/−*^ and *Deptor*^*−/−*^*;Pten*^*+/−*^ mice were sectioned and subjected to immunohistochemical staining with the indicated antibodies. The staining quantification was determined by IHC scoring using an IRS system from at least five random fields of prostate tissue sections. *p < 0.05; **p < 0.01; ***p < 0.001. **e** A model for DEPTOR suppression of prostate tumorigenesis

The viability of *Deptor*^*−/−*^ mice provided us an opportunity to study the role of Deptor in prostate tumorigenesis. It is well established that PTEN functions as a direct antagonist of PI3K activation and its genomic aberrations are one of the most commonly altered pathways in prostate cancer [37]. While homozygous loss of *Pten* causes embryonic lethality, heterozygous deletion is viable, but prone to the development of prostate cancer due to the moderate activation of PI3K/AKT/mTOR signaling [38]. We therefore tested the in vivo role of Deptor in prostate tumorigenesis triggered by *Pten* heterozygous loss. We crossed *Deptor*^*+/−*^ mice with *Pten*^*+/*^^−^ mice to generate compound mice with genotypes of *Deptor*^*+/+*^*;Pten*^*+/−*^ and *Deptor*^*−/*^^−^*;Pten*^*+/−*^ mice in a mixed 129/B6 background. Although genetic background was reported to affect the prevalence of prostate cancer [39], we found, however, that it has minimal effect on the formation of prostate tumor, if *Deptor* is deleted in combination with heterozygous *Pten* loss. Specifically, in all tested pairs of mice derived from three independent litters at the age of 13–16 months, the size of the prostates of *Deptor*^*−/−*^*;Pten*^*+/*^^−^ mice, compared with those of *Deptor*^*+/+*^*;Pten*^*+/−*^ mice, was much larger, and enlarged mass was covered with rich vascular anastomose, accompanied with hepatomegaly and focal tumor necrosis (Fig. 6a, Supplementary Fig. 7A). The H&E and immunohistochemical staining of tissue sections showed that prostate tumor tissues lost the glandular structures and were filled with many Ki67-positive proliferating cells (Fig. 6b, c and Supplementary Fig. 7B). Thus, *Deptor* loss significantly promoted prostate tumorigenesis triggered by *Pten* heterozygous loss.

We next determined whether mTORC1 and mTORC2 signals were activated in *Deptor*-null prostate tumors. Indeed, the phosphorylation of S6, 4E-bp1, and Akt, the downstream effectors of the mTORC1/2 pathways, were all significantly increased in the prostate tumors from *Deptor*^*−/−*^*;Pten*^*+/−*^ mice, as compared with those from *Deptor*^*+/+*^*;Pten*^*+/−*^ mice (Fig. 6d). Thus, *Deptor* deletion further activates mTORC1 and mTORC2 signals, which promotes prostate tumorigenesis.

## Discussion

In this study, using human prostate cancer tissues and cell culture, as well as mouse KO model, we showed that DEPTOR plays a tumor suppressive role in the prostate by inactivating both mTORC1 and mTORC2 signals. Our conclusion is supported by the following lines of evidence: (1) Reduced expression of DEPTOR mRNA and protein was seen in human prostate cancer tissues, which directly correlated with disease progression; (2) DEPTOR depletion promoted cell proliferation and survival via the activation of S6K1 and AKT; (3) Dual mTORC1/2 inhibitor Torin-1 abrogated the proliferation-promoting effect of DEPTOR depletion; (4) DEPTOR depletion promoted migration and invasion, which is likely caused by AKT-mediated Snail induction and β-catenin nuclear translocation; (5) AKT inhibitor MK-2206 abrogated enhanced migration and invasion by DEPTOR depletion; and (6) *Deptor* disruption promoted prostate tumorigenesis triggered by *Pten* heterozygous loss with activated mTORC1 and mTORC2 seen in resulting prostate tumor tissues.

Alterations in DEPTOR were reported in multiple human malignancies, including pancreatic cancer [40], lung cancer [41], colorectal cancer [42], hepatocellular carcinoma [43], and esophageal squamous cell carcinoma [44], using cell culture settings and/or human patient tissue samples. Given that DEPTOR is negatively regulated by mTOR signals, whereas mTOR signals are generally activated in human malignancies, it is not surprised that DEPTOR levels are frequently decreased in multiple malignancies [21]. However, it is unknown whether decreased DEPTOR plays a causal role or is merely a consequence of tumorigenesis.

In cell culture settings, opposite roles of DEPTOR have been reported, either acting as a tumor suppressor by inactivating mTORC1/2 [21, 40, 41, 44] or acting as an oncogene by relieving the feedback inhibition from S6K1 to PI3K, leading to AKT activation [21]. Interestingly, in our study, overactivation of S6K1 upon DEPTOR depletion in prostate cancer cells and mice tissues did not inactivate PI3K/AKT signal (Figs. 2c, 3c, f, 4g, h, 6d, and Supplementary Fig. 3C). We speculated that although S6K activation suppresses AKT, as often seen in many other types of cells [22], in prostate cancer cells with DEPTOR depletion, AKT activation by overactivated mTORC2 could neutralize or counteract S6K effect. Moreover, it has been reported previously that increased DEPTOR rendered cancer cells more resistant to chemotherapeutic drugs, which could be restored by AKT inactivation [45, 46], and DEPTOR overexpression correlates with poor prognosis of hepatocellular carcinoma and differentiated thyroid carcinoma patients [23, 24]. These conflicting findings suggest the role of DEPTOR in the regulation of tumorigenesis is rather complicated and likely cell/tissue context dependent. Thus, using *Deptor*-KO mouse models are the only way to clearly demonstrate the in vivo physiological role of DEPTOR in tumorigenesis and whether it is functioning as a tumor suppressor or an oncoprotein.

In this study, we successfully generated *Deptor-*KO mice, which were viable and fertile without any observable defects. The viability of *Deptor*^*−/−*^ mice provided us an opportunity to study the role of DEPTOR in tumorigenesis in combination with inactivation of a tumor suppressor or activation of an oncogene, such as *Pten* loss or Ras activation. To the best of our knowledge, this is the first case to explore the physiological role of DEPTOR in tumorigenesis using a *Deptor*-KO mouse model in combination with *Pten* loss. To investigate how the PTEN-PI3K-AKT-mTOR- DEPTOR axis regulates prostate tumorigenesis, we generated the *Deptor*^*−/−*^*;Pten*^*+/−*^ compound mice with partially elimination of the upstream inhibitor of the PI3K-AKT-mTOR axis (*Pten*^*+/−*^) and completely elimination of the downstream inhibition of mTOR activation (*Deptor*^*−/−*^). Indeed, we found that *Deptor* KO substantially accelerated mouse prostate tumorigenesis triggered by *Pten* insufficiency (Fig. 6 and Supplementary Fig. 7), indicating that Deptor plays a co-operative tumor suppressor role with Pten. Finally, our *Deptor*-KO model can be widely used to elucidate the physiological role of DEPTOR in tissue specific tumorigenesis.

What is the translational implication of this study? First, since DEPTOR is frequently downregulated in various cancer types, likely transcriptionally repressed by both mTORC1 and mTORC2 [21]. mTOR inhibitors should reactivate DEPTOR, particularly in prostate cancer with low DEPTOR expression; Second, DEPTOR is subjected to ubiquitylation by SCF^βTrCP^ E3 ubiquitin ligase for proteasome degradation [45–47], thus inhibition of SCF E3 by MLN4924 should cause DEPTOR accumulation [48]. Third, elucidation of the mechanism by which DEPTOR is downregulated in prostate cancer would provide a new strategy to reactivate DEPTOR to slow down the disease progression. Taken together, our study provides new directions for the treatment of prostate cancer patients by developing drugs that can restore DEPTOR levels.

In summary, our study supports the following working model. In the presence of DEPTOR, mTORC1/2 signals were inactivated, along with downstream S6K1 and AKT, leading to reduced proliferation and survival. Upon DEPTOR depletion during prostate tumorigenesis, mTORC1/2 signals were activated, followed by activation of S6K1 to promote proliferation, and activation of AKT for survival. AKT activation also induces Snail to promote EMT and β-catenin nuclear translocation to promote migration and invasion (Fig. 6e). Thus, the discovery of small molecules that can restore DEPTOR expression to inactivate mTOR/AKT signaling might be an attractive approach for future anti-prostate cancer therapy.

## Methods

### Cell lines and chemicals

Prostate cancer cell lines, DU145, 22RV1, RWPE, LNCap, and PC3, were obtained from American Type Culture Collection, and maintained in RPMI-1640 medium supplemented with 10% fetal bovine serum (FBS) and 1% penicillin–streptomycin at 37 °C in a humidified incubator with 5% CO_2_. Torin-1 (S2827) and MK-2206 (S1078) were purchased from Selleck. Rapamycin (HY-10219) was purchased from MedChemExpress.

### CRISPR/Cas9-mediated DEPTOR knockout

Single-guide RNA (sgRNA) against *Deptor* was subcloned into the plasmid pSpCas9(BB)-2A-Puro (PX459). The sgRNA targeted exon 2 in *DEPTOR*, leading to a frameshift mutation. DU145 and 22RV1 cells were transfected with the construct and selected with puromycin for 3 days, and single clones were picked under a microscope. The sequence of the sgRNA was as follows: DEPTOR-sgRNA: 5′-GGAGCTGGAGCGCATGGCTG-3′.

### siRNA-based DEPTOR knockdown

Cells were transfected with siRNA control or siDEPTOR in 60-mm dishes using Lipofectamine 2000 according to the manufacturer’s instructions (Invitrogen). After 48–72 h, the cells were split for proliferation, clonogenic survival, migration, and invasion assays. siCtrl: 5′-ATTGTATGCGATCGCAGAC-3′; siDEPTOR: 5′-GCCATGACAATCGGAAATCTA-3′.

### Retrovirus-based DEPTOR expression

Retroviruses expressing DEPTOR were produced by transfecting pBabe-DEPTOR, GAG, and VSV-G plasmids into Bosc 293 cells. DU145 cells were infected with retrovirus expressing mock vector, or DEPTOR, and then selected with puromycin for 7 days. Cells were then split for proliferation, clonogenic survival, and western blotting analyses.

### ATPlite-based cell proliferation and clonogenic survival assays

Cells were seeded in 96-well plates in triplicate at 1000 cells per well. Cell proliferation was evaluated by an ATPlite assay according to the manufacturer’s instructions (PerkinElmer) and results were expressed as the fold change compared with the control. For clonogenic survival assays, a total of 500 cells were seeded in 60-mm dishes for 7–14 days, which was followed by staining with Coomassie brilliant blue solution, and the dishes were photographed for colony counting (>50 cells in a colony). Clonogenic survival was expressed as the rate of colony formation (%) = colony number/500*100.

### Western blotting

Cells or tissues were lysed in lysis buffer with protease inhibitors and phosphatase inhibitors, which was followed by western blotting as previously described [49]. The following antibodies (Abs) were used: DEPTOR (11816, for human samples), p-AKT (S473) (4060), t-AKT (4691), p-S6K1 (T389) (9234), E-cadherin (14472), N-cadherin (13116), Vimentin (5741), Snail (3879), Slug (9585), ZEB1 (3396), LEF-1 (2230), Twist (46702), β-catenin (8480), PARP (9532), and caspase 3 (9662) (Cell Signaling Technology), DEPTOR (09–463, for mouse samples) (Millipore), t-S6K1 (sc-230) (Santa Cruz) and actin (A5441) (Sigma). The band density was quantified using the software Image J and expressed as the relative gray value (compared with the control), by arbitrarily setting the control value as 1.

### Wound healing assay

Confluent cell monolayers in six-well plates were scraped using a pipette tip to produce a wound. Detached cells were removed by washing with PBS. The initial wound at 0 h was photographed and set to 1. Cells were then incubated for up to 72 h in serum-free medium. The images of the wound were photographed at 24, 48, or 72 h after scratching. The width of the wound at multiple sites along the scratch was measured, and the relative width of the wound was expressed as the ratio between the average width of the wound at the indicated time points and the average width of the wound at 0 h.

### Transwell migration and invasion assays

Cells were maintained in serum-free medium for 12–18 h and then seeded in a 24-well plate upper chamber insert at 5 × 10^4^ per chamber in serum-free medium, with medium containing 10% FBS under the insert. For the invasion assay, Matrigel matrix was added to the upper chamber insert. After incubating for 12–24 h, cells were fixed and stained with 0.05% crystal violet. Cells on the upper surface of the membrane were then removed with a cotton swab. The migrated or invasive cells that were attached to the bottom of the membrane were photographed. The number of migratory or invasive cells in at least five random fields per chamber insert was counted.

### Nuclear and cytoplasm fractionation

Cells were harvested and lysed in buffer A (10 mM HEPES, pH 7.9, 20% glycerol, 10 mM KCl, 1.5 mM MgCl_2_, 0.5 mM DTT, and 0.5% NP-40) containing protease inhibitors, which was followed by centrifugation at 2000 × *g* for 2 min at 4 °C. The supernatants were collected as the cytoplasmic fractions. The nuclear pellets were washed with buffer A and then lysed in buffer B (20 mM HEPES, pH 8.0, 25% glycerol, 420 mM NaCl, 1.5 mM MgCl_2_, 0.2 mM EDTA, 0.5 mM DTT, 0.1% NP-40) containing protease inhibitors. After centrifugation at 13,600 rpm for 10 min at 4 °C, the supernatants were saved as the nuclear fractions.

### Generation of Deptor knockout mice

The strategy used for the *Deptor*^*fl/fl*^ conditional KO mouse that targeted exons 6 and 7, which were flanked with FRT and loxP sites, is shown in Supplementary Fig. 6A. A linearized targeting vector was electroporated into embryonic stem (ES) cells and selected by G418. Homologous integration clones were identified by PCR and Southern blotting. *Deptor*-targeted mouse ES cells were injected into C57BL/6 blastocysts to generate chimeras. Male chimeras were bred with Black Swiss females, and agouti offspring were genotyped by PCR. The floxed *Deptor* mice were created after deleting the neomycin resistance cassette by crossing frt-flanked *Deptor* mice with the recombinase flippase (Flp) transgenic mice. *Deptor*-KO mice were generated by crossing floxed Deptor mice with EIIα-Cre transgenic mice. For animal study, all procedures were approved by the University of Michigan Committee on Use and Care of Animals. Animal care was provided in accordance with the principles and procedures outlined in the National Research Council Guide for the Care and Use of Laboratory Animals.

### PCR-based genotyping and RT-PCR analysis

Genomic DNA was isolated from mouse tail tips by lysing in TNES lysis buffer (10 mM Tris-HCl, pH 7.5, 400 mM NaCl, 100 mM EDTA, and 0.6% SDS) containing proteinase K. Mice were genotyped using the following primers to detect Deptor deletion (500 bp) and WT (150 bp): Primer 1: 5′-TGGAGAGCAACTGGGGAAGA-3′; Primer 2: 5′-CTACGGGACCTCACCGAGAA-3′; Primer 3: 5′-AACAGGCATCTTTATCCCATCA-3′. For RT-qPCR, the sequences of the primer sets are as follows: Snail, 5′-AGCTGCAGGACTCTAATCCAGAGT′ (forward) and 5′-CGTGTGGCTTCGGATGTGC′ (reverse); GAPDH, 5′-AGGGCATCCTGGGCTACAC-3′ (forward) and 5′-GCCAAATTGGTTGTCATACCAG-3′ (reverse).

### Human prostate tissue microarray and immunohistochemistry

Human prostate tissue microarrays consisting of 59 pairs of tumors and adjacent normal tissues, and another 30 tumor samples from prostate cancer patients were obtained from Outdo Biotech Company (Shanghai, China). For IHC, the arrays were stained with anti-DEPTOR antibodies, followed by counterstaining with hematoxylin. The slides were then scanned by an Aperio Whole Slide Scanner and observed using Aperio ImageScope software. For quantitative evaluation, tissues in at least five random fields of each sample were photographed at ×20 magnification and were analyzed using an immunoreactive score as the IHC scoring scheme for DEPTOR expression. Stained tissues were classified into four groups according to the staining intensity: negative (0), weak (1), moderate (2), and strong (3). Depending on the percentage of positive cells, the proportion score of DEPTOR expression was classified as follows: 0, 0%; 1, ≤10%; 2, 11–50%; 3, 51–80%; and 4, ≥81%. The total score was calculated by multiplying the proportion score by the intensity score [50]. Sections of mouse prostate tissues that were 5 μm thick were stained with hematoxylin and eosin, or the following antibodies: DEPTOR (11816), Ki67 (12202), p-S6 (S235/236) (4858), p-4E-bp1 (T37/46) (2855), and p-Akt (S473) (4060) (Cell Signaling Technology), and then photographed under a microscope or scanned by an Aperio Whole Slide Scanner.

### Statistical analysis

The data from three independent experiments are presented as the mean ± SEM and were analyzed using GraphPad Prism 5. The Wilcoxon rank sum test was utilized to assess the DEPTOR expression in tumors and corresponding tumor-adjacent tissues. Two-sided Student’s *t*-test was performed with SPSS 20.0 (IBM Armonk) to compare the parameters between groups. *p* < 0.05 was considered statistically significant.

## Supplementary information

Supplemental information
