# Supplementary material for: DEPTOR is an in vivo tumor suppressor that inhibits prostate tumorigenesis via the inactivation of mTORC1/2 signals
Source: Oncogene. 2019 Nov 4;39(7):1557–71. doi: 10.1038/s41388-019-1085-y (PMC7018663; doi:10.1038/s41388-019-1085-y)

Supplemental information

Supplemental figure legends

Figure S1. The specificity of DEPTOR antibody used in immunohistochemistry (IHC) assay.

A. Human prostate cancer tissues were immunohistochemically stained using DEPTOR antibody with or without blocking peptide. B. sgCtrl and sgDEPTOR 22RV1 cells were stained using DEPTOR antibody based on IHC procedure. C. Xenografts developed from MDA-MB361 shGFP and shDEPTOR cells were stained using DEPTOR antibody.

Figure S2. The expression of DEPTOR, S6K1 and AKT in multiple prostate cancer cell lines.

Cells were harvested and subjected to Western blotting using the indicated antibodies. Human prostate cancer DU145 and 22RV1 cells with high expression of DEPTOR and low phosphorylation levels of S6K1 and AKT were selected for study.

Figure S3. DEPTOR knockdown promotes cell proliferation and clonogenic survival via the activation of AKT and S6K1 in prostate cancer cells.

A. Cells were transfected with the indicated siRNA oligos for 48-72 h and then split for the ATPlite-based proliferation assay. The mean ± SEM are shown from three independent experiments; n=3; *p < 0.05; **p < 0.01. B. A total of 500 cells transfected with the indicated siRNA oligos were seeded in triplicate in 60-mm dishes. After 7-14 days, cells were stained and photographed (left), and the numbers of colonies were counted (right). The mean ± SEM are shown from three independent experiments; n=3; *p < 0.05; **p < 0.01. C. Cells were transfected with the indicated siRNA oligos, followed by Western blotting using the indicated antibodies.

Figure S4. The phosphorylation of S6K1 and AKT was blocked by Torin-1 at different concentrations.

DU145 cells were treated with various doses of Torin-1 for 12 h and then harvested for Western blotting using the indicated antibodies. The concentration of 100 nM was chosen for rescue assays in DU145 cells. LEX: longer exposure.

Figure S5. DEPTOR knockdown enhances cell migration and invasion in

prostate cancer cells.

A. Cells were transfected with the indicated siRNA oligos, followed by Western blotting using the indicated antibodies to determine the effect of DEPTOR silencing. B-D. Cell migration was examined using transwell migration assay (B) and wound healing assay (C, D). Representative images of migratory cells (left panel of B) and wound healing at indicated time points (left panels of C and D) were photographed. The number of migratory cells was counted in five random fields per chamber insert (right panel of B). The relative wound width was calculated as the ratio between the width of the wound at different time points and the width of wound at 0 h (right panels of C and D). The mean ± SEM are shown from three independent experiments; n=5; **p < 0.01; ***p < 0.001. E. Cell invasion was determined using a transwell invasion assay with Matrigel. The mean ± SEM are shown from three independent experiments; n=5; ***p < 0.001.

Figure S6. Generation of *Deptor*$^{-/-}$ mice.

A. The strategy used to target exons 6 and 7 of the *Deptor* allele. B. PCR analysis of genomic DNA of tails from *Deptor*$^{+/+}$, *Deptor*$^{+/-}$ and *Deptor*$^{-/-}$ mice. C. The brian and liver tissues from three individual mice with each of the indicated genotypes were harvested for Western blotting to verify Deptor deletion. D. Disruption of Deptor did not cause embryonic and postnatal death. The numbers of mice over 5 weeks of age with different *Deptor* genotypes were summarized. E. A *Deptor*$^{-/-}$ mouse (right) and its *Deptor*$^{+/+}$ littermate (left) at the age of 3 weeks were photographed. F. The bodies of the littermate female and male mice with the indicated genotypes at the age of 3 weeks were weighed. The mean ± SD are shown.

Figure S7. *Deptor* knockout induces prostate tumorigenesis *in vivo*.

A. The morphology of littermate *Deptor*$^{+/+}$*; Pten*$^{+/-}$ and *Deptor*$^{-/-}$*; Pten*$^{+/-}$ mice from three paired mice. B. H&E staining of prostate tissues from paired mice (A).

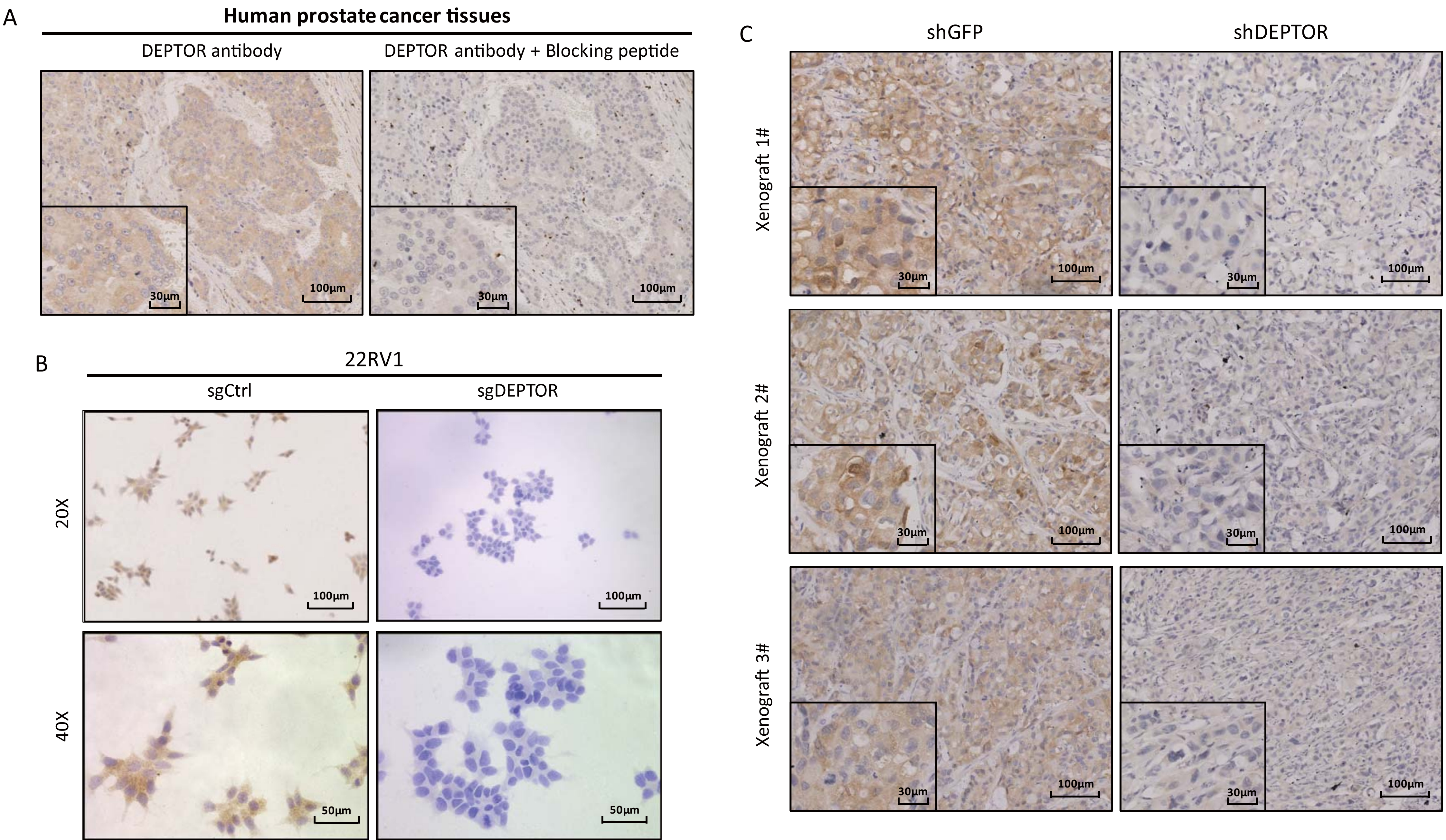
A
Human prostate cancer tissues
DEPTOR antibody
DEPTOR antibody + Blocking peptide
30µm
100µm
B
22RV1
sgCtrl
sgDEPTOR
20X
40X
100µm
50µm
C
shGFP
shDEPTOR
Xenograft 1#
Xenograft 2#
Xenograft 3#
30µm
100µm

Chen et al. Figure S2

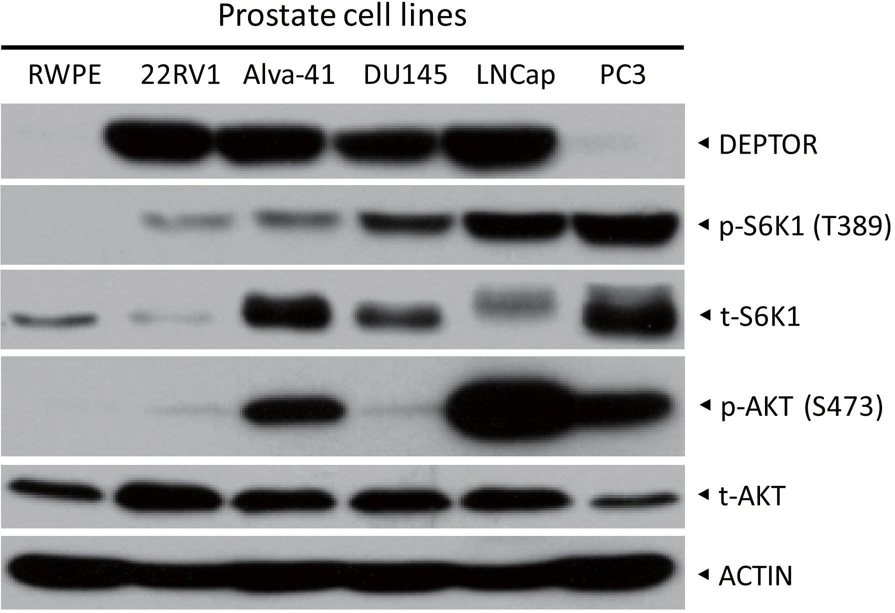

Chen et al. Figure S3

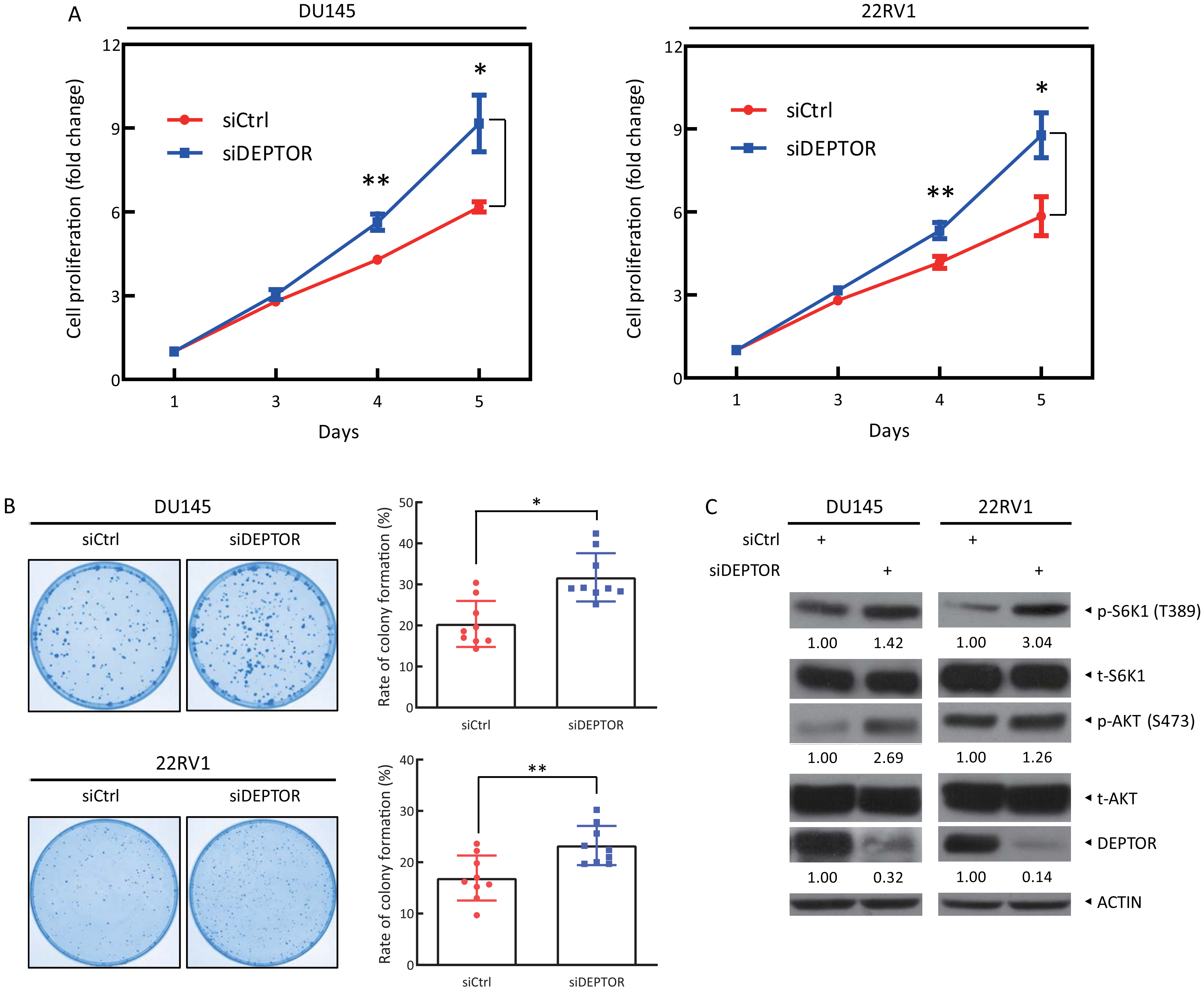

Chen et al. Figure S4

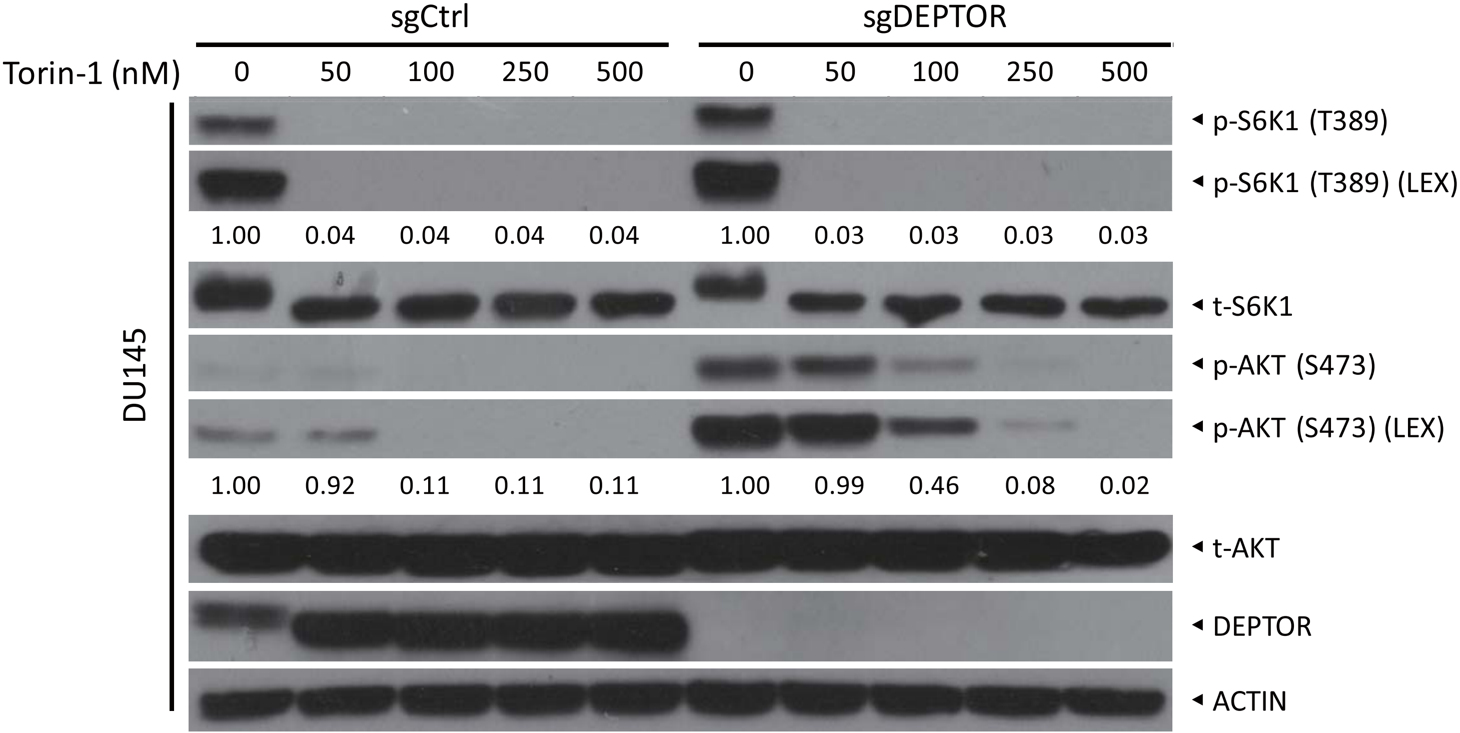

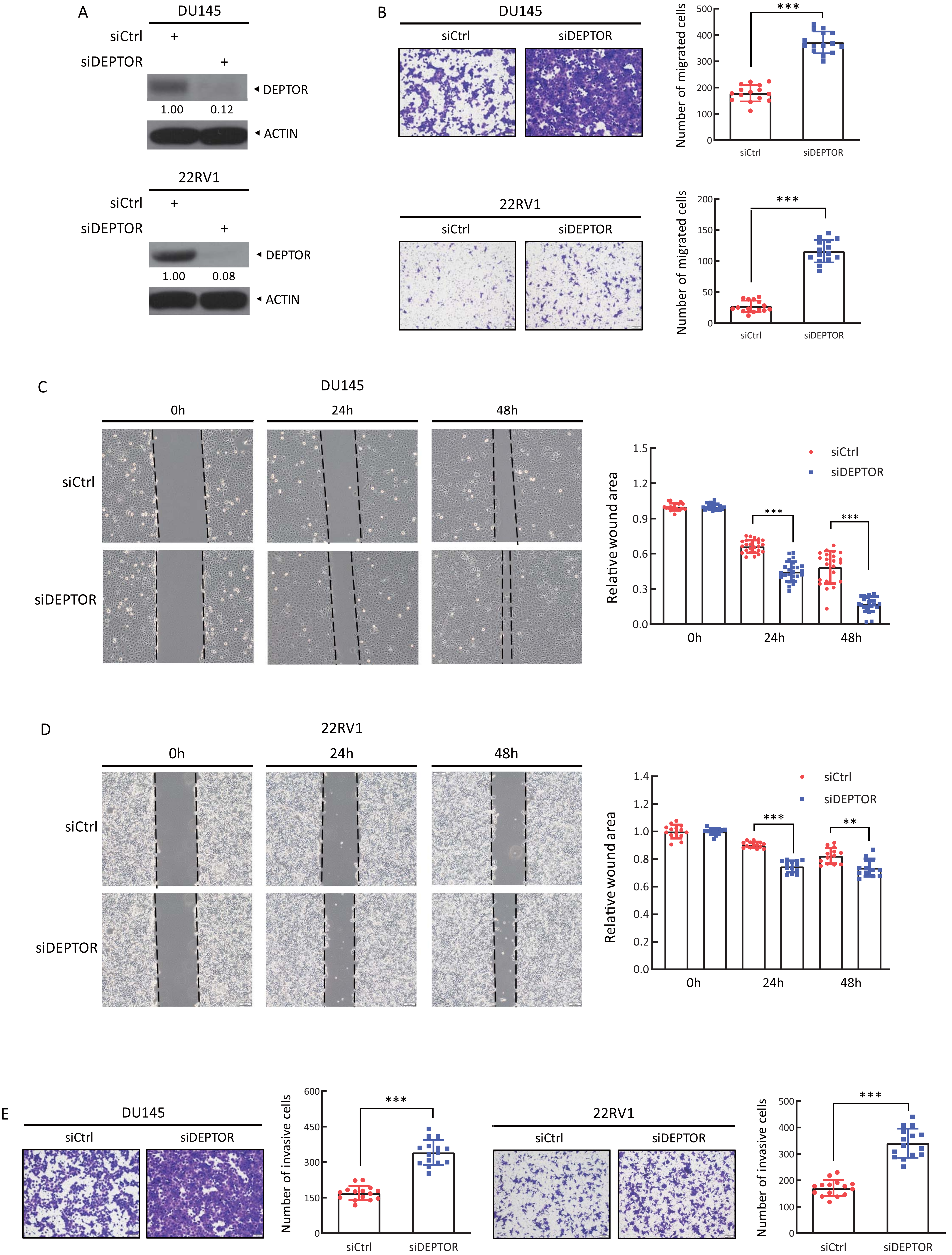
A
DU145
siCtrl +
siDEPTOR +
DEPTOR
1.00 0.12
ACTIN
22RV1
siCtrl +
siDEPTOR +
DEPTOR
1.00 0.08
ACTIN
B
DU145
siCtrl siDEPTOR
Number of migrated cells
***
siCtrl siDEPTOR
22RV1
siCtrl siDEPTOR
Number of migrated cells
***
siCtrl siDEPTOR
C
DU145
0h 24h 48h
siCtrl
siDEPTOR
Relative wound area
siCtrl
siDEPTOR
***
***
0h 24h 48h
D
22RV1
0h 24h 48h
siCtrl
siDEPTOR
Relative wound area
siCtrl
siDEPTOR
***
**
0h 24h 48h
E
DU145
siCtrl siDEPTOR
Number of invasive cells
***
siCtrl siDEPTOR
22RV1
siCtrl siDEPTOR
Number of invasive cells
***
siCtrl siDEPTOR

A B C

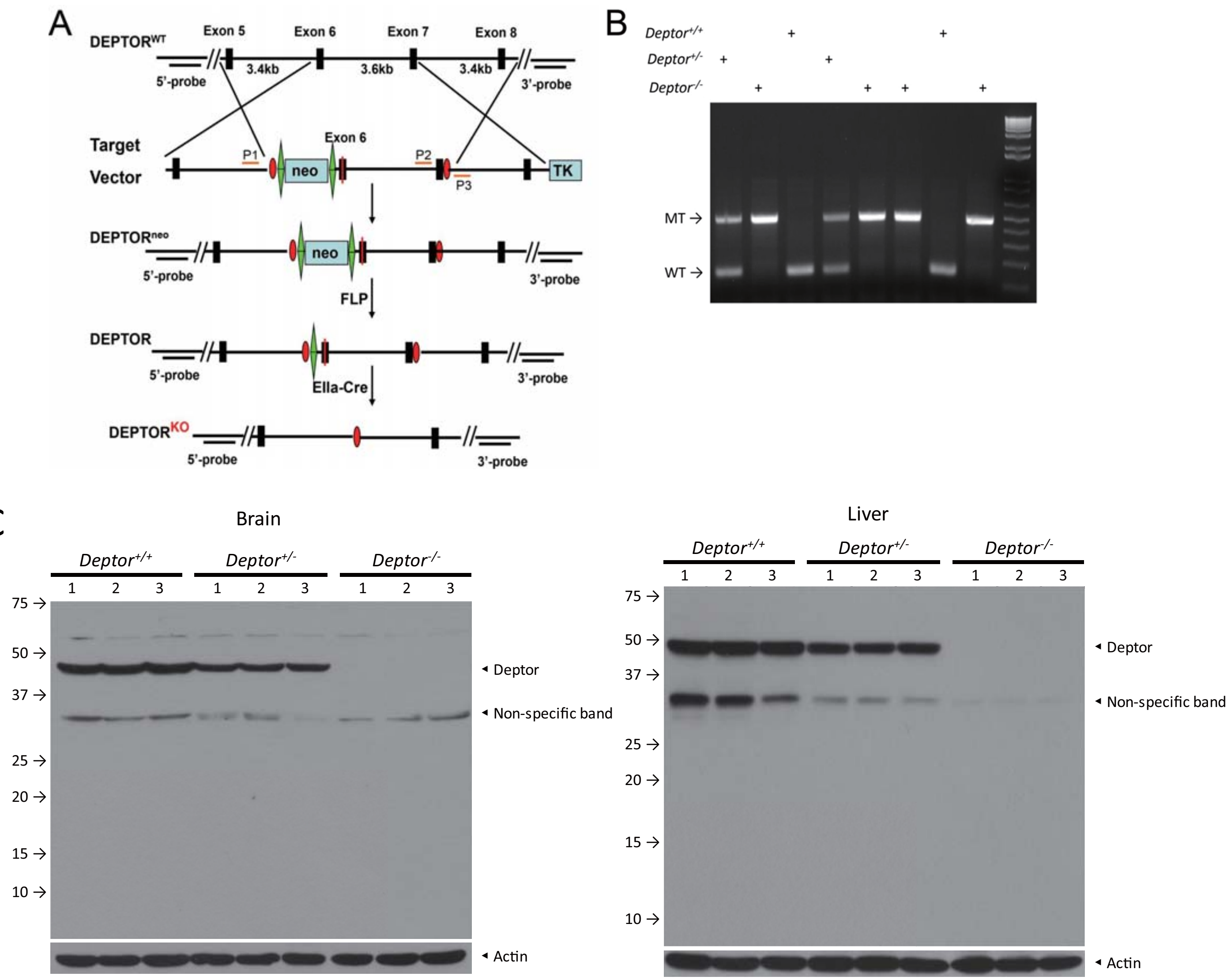

D

	Deptor+/+	*Deptor+/-*	*Deptor-/-*	Total
No. of mice (>5wks)	90	181	85	356
Expected ratio	1/4	1/2	1/4	

E

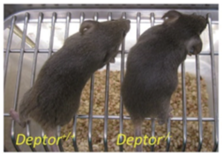

F

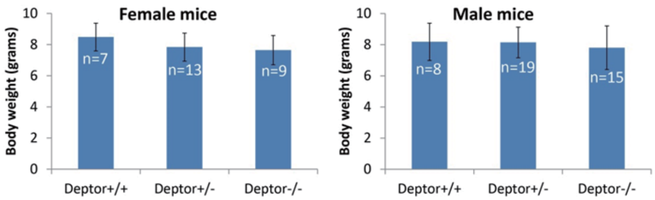

Chen et al. Figure S7

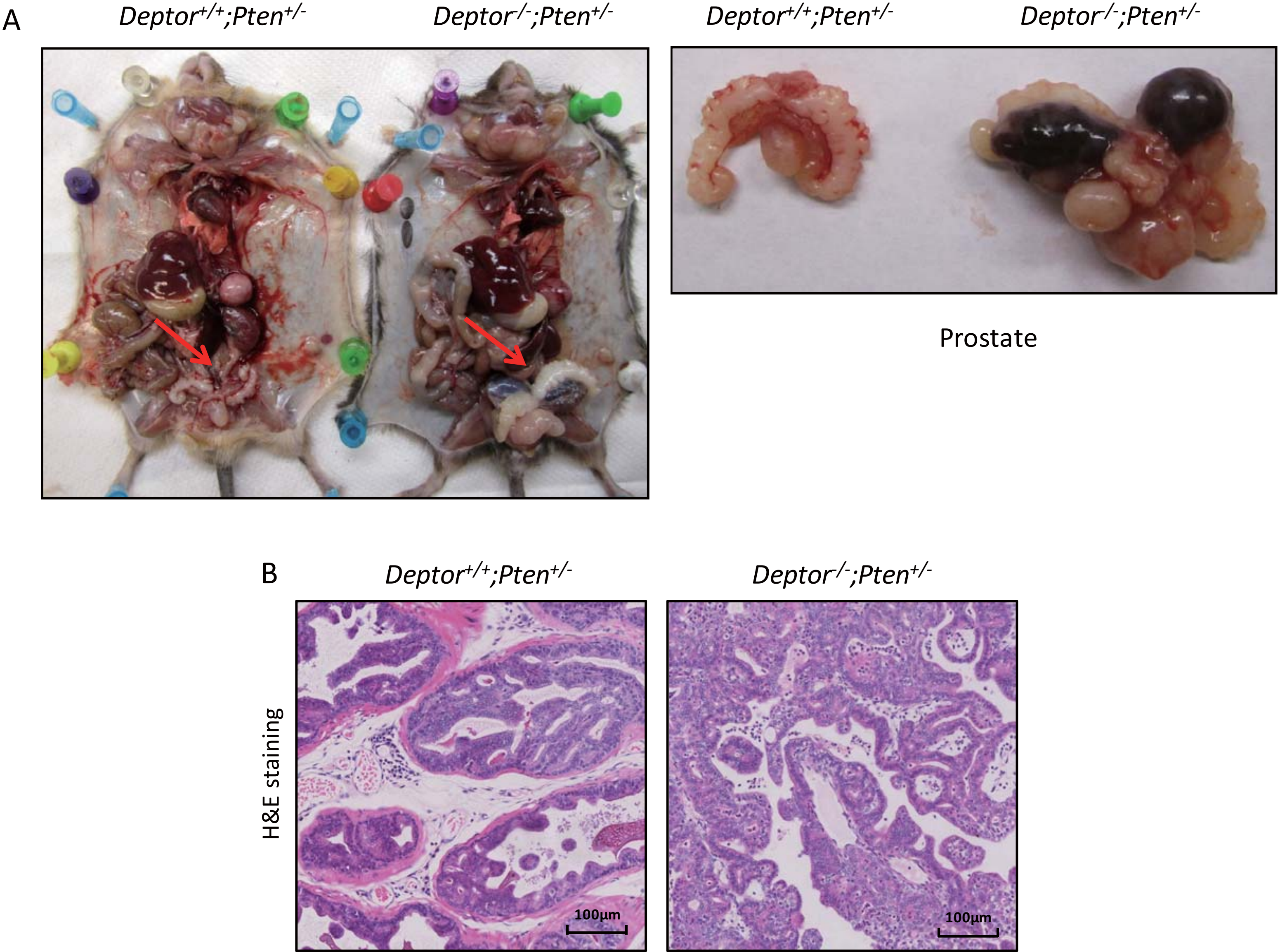

Supplement: Supplementary file 1 — Supplemental information [file 41388_2019_1085_MOESM1_ESM.pdf]
